# CO_2_ Methanation over Ni Catalysts Supported on Pr-Doped CeO_2_ Nanostructures Synthesized via Hydrothermal and Co-Precipitation Methods

**DOI:** 10.3390/nano15131022

**Published:** 2025-07-01

**Authors:** Anastasios I. Tsiotsias, Nikolaos D. Charisiou, Aasif A. Dabbawala, Aseel G. S. Hussien, Victor Sebastian, Steven J. Hinder, Mark A. Baker, Samuel Mao, Kyriaki Polychronopoulou, Maria A. Goula

**Affiliations:** 1 Laboratory of Alternative Fuels and Environmental Catalysis (LAFEC), Department of Chemical Engineering, University of Western Macedonia, 50100 Kozani, Greece; antsiotsias@uowm.gr; 2 Department of Mechanical Engineering, Khalifa University of Science and Technology, Abu Dhabi P.O. Box 127788, United Arab Emirates; aasif.dabbawala@ku.ac.ae (A.A.D.); aseel.ghussien@ku.ac.ae (A.G.S.H.); samuel.mao@ku.ac.ae (S.M.); kyriaki.polychrono@ku.ac.ae (K.P.); 3 Center for Catalysis and Separations, Khalifa University of Science and Technology, Abu Dhabi P.O. Box 127788, United Arab Emirates; 4 Department of Chemical Engineering and Environmental Technology, Universidad de Zaragoza, Campus Río Ebro-Edificio I+D, 50018 Zaragoza, Spain; victorse@unizar.es; 5 Instituto de Nanociencia y Materiales de Aragón (INMA), Universidad de Zaragoza–CSIC, c/María de Luna 3, 50018 Zaragoza, Spain; 6 Networking Research Center on Bioengineering, Biomaterials and Nanomedicine, CIBERBBN, 28029 Madrid, Spain; 7 The Surface Analysis Laboratory, Faculty of Engineering and Physical Sciences, University of Surrey, Guildford GU2 4DL, UK; s.hinder@surrey.ac.uk (S.J.H.); m.baker@surrey.ac.uk (M.A.B.); 8 Centre for Research & Technology Hellas (CERTH), Chemical Process and Energy Resources Institute (CPERI), 52 Egialias Str., 15125 Athens, Greece; 9 School of Science and Technology, Hellenic Open University, Parodos Aristotelous 18, 26335 Patras, Greece

**Keywords:** CO_2_ methanation, synthesis method, Pr-doped CeO_2_, hydrothermal, co-precipitation, nanostructures

## Abstract

The synthesis method of the Pr-doped CeO_2_ catalyst support in Ni/Pr-CeO_2_ CO_2_ methanation catalysts is varied by changing the type/basicity of the precipitating solution and the hydrothermal treatment temperature. The use of highly basic NaOH as the precipitating agent and elevated hydrothermal treatment temperature (100 or 180 °C) leads to the formation of structured Pr-doped CeO_2_ nanorods and nanocubes, respectively, whereas the use of a mildly basic NH_3_-based buffer in the absence of hydrothermal treatment (i.e., co-precipitation) leads to an unstructured mesoporous morphology with medium-sized supported Ni nanoparticles. The latter catalyst (Ni/CP_NH3) displays a high surface area, high population of moderately strong basic sites, high oxygen vacancy population, and favorable Ni dispersion. These properties lead to a higher catalytic activity for CO_2_ methanation (75% CO_2_ conversion and 99% CH_4_ selectivity at 350 °C) compared to the catalysts with structured nanorod and nanocube support morphologies, which are found to contain a significant amount of leftover Na from the synthesis procedure that can act as a catalyst inhibitor. In addition, the best-performing Ni/CP_NH3 catalyst is shown to be highly stable, with minimal deactivation during time-on-stream operation.

## 1. Introduction

The swift increase in anthropogenic CO_2_ emissions risks disrupting the Earth’s climate, since CO_2_ functions as a greenhouse gas leading to a steep rise in the atmospheric temperature [1,2]. To keep its concentration in check, it is crucial to develop effective carbon capture and storage, as well as carbon capture and utilization technologies, the latter of which can result in the generation of value-added products from waste CO_2_ streams [3,4,5]. On the other hand, the fluctuating nature of renewable energy production necessitates long-term energy storage solutions, which can be achieved through chemical energy storage in the form of “green” hydrogen that is produced through electrolysis [6]. However, hydrogen has a low volumetric energy density, presenting challenges for its effective storage and transportation [7]. To this end, the produced green hydrogen can be used to hydrogenate the captured CO_2_ to generate CH_4_ (synthetic natural gas), which presents a much higher energy density, as well as easier storage and transportation options, via the CO_2_ methanation reaction (Equation (1)) [8,9,10].

CO_2_ + 4H_2_ → CH_4_ + 2H_2_O (1)

Noble metal catalysts like Rh and Ru have demonstrated significant catalytic activity for this particular reaction [9,10,11]. However, their prohibitively high cost remains a considerable limitation, leading to the widespread use of alternative, transition metal catalysts, particularly Ni-based ones [12,13]. Ni catalysts supported on CeO_2_-based supports are known for their superior CO_2_ methanation catalytic activity when compared to those supported on other metal oxide supports (e.g., Al_2_O_3_, SiO_2_, or ZrO_2_), which is ascribed to the rich defect chemistry and high oxygen vacancy population/oxygen mobility of the CeO_2_ lattice, facilitating the rapid conversion and removal of intermediate species [13,14,15]. The doping of CeO_2_ with trivalent cations, like La^3+^ [16], Sm^3+^ [17], and Pr^3+^ [18,19,20], has also been shown to significantly enhance the oxygen vacancy population (to charge-balance the extrinsic substitutional defects) and improve the CO_2_ methanation catalytic activity. Particularly, in our previous work [20], we found that the Pr-doping of the CeO_2_ support at 10 mol% can provide the maximum promoting effect for Ni-supported CO_2_ methanation catalysts.

In recent years, many research studies have focused on the hydrothermal synthesis of CeO_2_-based supports with varying morphologies and exposed crystalline facets, since hydrothermal treatment is typically employed to generate specific nanostructures for the CeO_2_-based oxides [21,22,23,24,25,26,27]. The hydrothermal synthesis utilizing a highly basic/concentrated NaOH solution as the precipitating agent has been frequently used for this purpose, with variations in the hydrothermal treatment temperature leading to the formation of nanorods, nanocubes, or other metal oxide support nanostructures [21,22,24,25,26]. For example, Hashimoto et al. [21] demonstrated that a Ni catalyst supported on hydrothermally prepared CeO_2_ with nanorod morphology exhibited a higher catalytic activity when compared to those supported on CeO_2_ with nanocube and nano-octahedral morphology, which was attributed to the enhanced surface oxygen reactivity of the (110) facet of crystalline CeO_2_ in the nanorods. Similar findings have also been reported by Bian et al. [22] and Ma et al. [23]. Conversely, according to Jomjaree et al. [24], Ni supported on CeO_2_ nanopolyhedrons and, according to Bian et al. [25], Ni supported on CeO_2_ nanoparticles, both prepared using more diluted/less basic NaOH precipitating solutions, were superior compared to Ni supported on nanorod and nanocube CeO_2_ support morphologies. The utilization of precipitating solutions with a weaker basicity during hydrothermal synthesis remains considerably less common in the literature. Furthermore, co-precipitation synthesis for the CeO_2_-based support, which often proceeds similarly to hydrothermal synthesis (but without the hydrothermal treatment step at an autoclave), has also been employed in a number of studies [28,29,30], but it generally receives significantly less attention compared to the hydrothermal synthesis, and these similar preparation techniques are rarely compared with each other.

In this work, we perform a comparative study by altering the support synthesis method in Ni-based catalysts via the variation of two synthesis parameters: (i) the basicity of the precipitating solution (highly basic NaOH vs. mildly basic NH_3_-based buffer) and (ii) the hydrothermal treatment temperature (100 °C, 180 °C, or room temperature, i.e., co-precipitation). We aim to investigate the potential advantage of hydrothermal synthesis compared to the simpler co-precipitation synthesis, and assess the effect of the precipitating agent, thus the need to use either a higher or a lower basicity precipitating solution. The doping of the CeO_2_ support with 10 mol% Pr was performed in this work to enhance the activity of the corresponding catalysts, based on the results of our prior work [20].

It is found that the use of a mildly basic NH_3_-based buffer in the absence of hydrothermal treatment (co-precipitation) leads to an unstructured mesoporous support morphology that provides a significantly higher catalytic activity during CO_2_ methanation. This can be attributed to favorable physicochemical properties such as a high surface area, high basic site population of moderate strength, high oxygen vacancy population, suitable Ni dispersion, and the absence of leftover Na that can act as a catalyst inhibitor. Therefore, we conclude that the rather simpler co-precipitation support synthesis with a mildly basic precipitating agent can yield better catalytic results, thus eliminating the need to employ hydrothermal treatment and utilize highly basic NaOH during the catalyst synthesis.

## 2. Materials and Methods

### 2.1. Synthesis Methods

Pr-doped CeO_2_ nanostructures with 10 mol% Pr nominal composition, or Ce_0.9_Pr_0.1_O_2−δ_ (“δ” is used for the extent of oxygen deficiency), were synthesized via several hydrothermal and co-precipitation synthesis methods by varying the precipitating agent, i.e., the basicity of the precipitating solution during the hydrothermal/co-precipitation synthesis, and the temperature of the hydrothermal treatment (Table 1).

When NaOH was used as the precipitating agent (high basicity of the precipitating solution, pH > 14), the following procedure was followed: At first, Ce(NO_3_)_3_·6H_2_O (Aldrich, St. Louis, MO, USA, 99%) and Pr(NO_3_)_3_·6H_2_O (Aldrich, 99.9%) in calculated amounts were dissolved in 50 mL of d-H_2_O in a beaker under stirring. In another beaker, NaOH (Fluka, Charlotte, NC, USA, K ≤ 0.02%, pellets) was added in 150 mL of d-H_2_O, so that the final concentration of NaOH (200 mL final solution) would be 10 M. The two solutions were then mixed together, and the final mixture was stirred for another 30 min and then transferred to a 300 mL Teflon-lined stainless-steel autoclave. Two hydrothermal treatment protocols were used: In the first one, the temperature was increased up to 100 °C (NaOH_100), and in the second one up to 180 °C (NaOH_180). In both cases, the mixture remained at that temperature for 24 h. A further co-precipitation protocol was performed, where the final mixture was kept at room temperature for 24 h without undergoing any hydrothermal treatment (CP_NaOH).

When an NH_3_-based buffer (NH_3_/(NH_4_)_2_CO_3_) was used as the precipitating agent (low basicity of the precipitating solution, pH = 9), the following procedure was followed: At first, calculated amounts of the metal nitrates of Ce and Pr were dissolved in 100 mL of d-H_2_O under stirring. The pH was then adjusted to 9 via the dropwise addition of a buffer solution of 3 M NH_3_/(NH_4_)_2_CO_3_. The volume was then increased to 200 mL (pH remaining at 9) via the further addition of d-H_2_O and some buffer solution. The final mixture was stirred for 30 min and then transferred to a 300 mL Teflon-lined stainless-steel autoclave. Two hydrothermal treatment protocols were used: In the first one, the temperature was increased up to 100 °C (NH3_100), and in the second one up to 180 °C (NH3_180). In both cases, the mixture remained at that temperature for 24 h. A further co-precipitation protocol was performed, where the final mixture was kept at room temperature for 24 h without undergoing any hydrothermal treatment (CP_NH3).

In all cases, after the 24 h treatment at either room temperature, 100 °C, or 180 °C, the final mixtures were centrifuged, the recovered solids were then washed thoroughly with d-H_2_O and once with ethanol, then dried at 70 °C overnight, and afterwards calcined at 500 °C for 4 h under static air to prepare the corresponding Pr-doped CeO_2_ support oxides with varying nanostructures.

Wet impregnation was then used to introduce the catalytically active Ni phase. At first, Ni(NO_3_)_2_·6H_2_O (Fluka, 97%), for a final Ni loading of 10 wt%, was added in 100 mL of d-H_2_O. The metal oxide support powder was then added to the solution under stirring. Afterwards, the water was removed in a rotary evaporator at 72 °C and the leftover slurry was overnight dried at 90 °C, and then calcined at 400 °C for 4h to obtain the calcined catalysts (NiO/Support). To prepare the corresponding reduced catalysts (Ni/Support), the calcined ones were reduced under H_2_ flow at 500 °C for 1 h.

### 2.2. Characterization Techniques

X-ray diffraction (XRD) was performed on a MiniFlex II Rigaku powder diffractometer (Tokyo, Japan), using a Cu-K_α1_ radiation source at 30 kV and 20 mA. To calculate the crystallite sizes of each phase, the Scherrer equation was applied on the strongest reflection.

N_2_ physisorption (adsorption/desorption) was carried out on a 3Flex instrument (Micromeritics, Norcross, GA, USA) at 77K. The specific surface area (SSA, m^2^/g) was determined via the multi-point Brunauer–Emmet–Teller (BET) method for 0.05 < P/P_0_ < 0.20, and the pore size distribution (PSD) via the Barrett–Joyner–Halenda (BJH) theory.

H_2_-temperature programmed reduction (H_2_-TPR), CO_2_-temperature programmed desorption (CO_2_-TPD), and H_2_-temperature programmed desorption (H_2_-TPD) were all performed on an Autochem 2920 instrument (Micromeritics). For H_2_-TPR on the calcined catalysts, the samples were first treated under 20% O_2_/He at 500 °C for 2 h and then cooled down to ambient temperature. Afterwards, 10% H_2_/Ar was passed through the materials during the temperature ramp (30 °C/min). For CO_2_-TPD on the reduced catalysts, the samples were first treated under H_2_ flow at 500 °C for 2 h. Afterwards, 10% CO_2_/He was flown at room temperature for the CO_2_ adsorption. The temperature was then increased with a 30 °C/min ramp under He flow. For H_2_-TPD on the reduced catalysts, the samples were first treated under H_2_ flow at 500 °C for 2 h, and then purged under Ar flow. Afterwards, 10% H_2_/Ar was flown at room temperature for the H_2_ adsorption. The temperature was then increased with a 30 °C/min ramp under Ar flow. In all cases, the thermal conductivity detector (TCD) signal was continuously recorded during the temperature ramps.

Raman spectroscopy was carried out on a Horiba Scientific LabRAM HR Evolution Raman spectrometer (Lille, France) that had a 633 nm laser and a research grade optical microscope with various lenses, featuring manual sample positioning with planar and depth scans. A total of 5 scans were taken for each sample, with a 20 s acquisition time.

X-ray photoelectron spectroscopy (XPS) was performed on a ThermoFisher Scientific K-Alpha+ instrument (East Grinstead, UK) using a monochromated Al K_a_ X-ray source (1486.6 eV) with a 400 μm radius X-ray spot. In total, 200 eV pass energy was employed for the survey spectra and 50 eV for the core level spectra (higher resolution). Instrument modified sensitivity factors were used for quantification. The adventitious carbon C1s peak at 285.0 eV was used for charge referencing.

Lastly, transmission electron microscopy (TEM) was performed on a G2 20 S-Twin FEI Tecnai microscope (Hillsboro, OR, USA) featuring a LaB_6_ electron source and a “SuperTwin^®^” objective lens that allows point-to-point resolution of 0.24 nm. High-angle annular dark field scanning transmission electron microscopy (STEM–HAADF) along with energy dispersive X-ray spectroscopy (EDS) analysis were carried out on an Analytical Titan (FEI) field emission gun microscope (300 kV), featuring a Cs-probe that allows electron probe formation of 0.09 nm (CEOS, Heidelberg, Germany).

### 2.3. Catalytic Testing

Catalytic testing was carried out at ambient pressure in a fixed-bed quartz reactor (I.D. = 0.9 cm), with a similar procedure as that described in Ref. [31]. All catalysts were previously reduced in situ for 1 h at 500 °C under H_2_ flow. The catalytic performance was evaluated using three experimental protocols (#1, #2, and #3) under a continuous-flow gas feed of 10% CO_2_, 40% H_2_, balance Ar, and 100 mL/min total flow.

In short, under Experimental Protocol #1, the catalytic activity was studied as a function of reaction temperature under a relatively low WHSV of 25,000 mL g_cat_^−1^ h^−1^. The temperature of the reactor was gradually increased in 50 °C steps from 200 °C up to 450 °C.

Under Experimental Protocol #2, a higher WHSV of 100,000 mL g_cat_^−1^ h^−1^ was employed, along with additional temperature steps every 10 °C from 250 °C up to 350 °C. The activation energy was calculated via this experimental protocol, making the assumption of pseudo-first order reaction kinetics and for low values of CO_2_ conversion (<20%), to negate the influence of mass transfer.

Under Experimental Protocol #3, the catalyst was evaluated regarding its stability during time-on-stream testing for 24 h at a constant temperature of 350 °C (WHSV = 25,000 mL g_cat_^−1^ h^−1^, as in Experimental Protocol #1). The spent catalyst was then reloaded in the reactor for an additional 24 h time-on-stream testing.

The gases exiting the reactor were analyzed online by a gas chromatography analysis system, as described in Ref. [18]. Besides CH_4_, CO was the sole hydrogenation by-product detected. Deviations calculated for the carbon balance were limited to ±3%. The following Equations (2)–(4) were used in order to calculate the reaction metrics (CO_2_ conversion, CH_4_ selectivity, and CH_4_ yield):

(2) $$X_{{\mathrm{CO}}_{2}}\;(\%)\;=\frac{C_{{\mathrm{CH}}_{4}}^{\mathrm{out}}+C_{\mathrm{CO}}^{\mathrm{out}}}{C_{{\mathrm{CO}}_{2}}^{\mathrm{out}}+C_{{\mathrm{CH}}_{4}}^{\mathrm{out}}+C_{\mathrm{CO}}^{\mathrm{out}}}\;\cdot 100$$

(3) $$S_{{\mathrm{CH}}_{4}}\;(\%)\;=\frac{C_{{\mathrm{CH}}_{4}}^{\mathrm{out}}}{C_{{\mathrm{CH}}_{4}}^{\mathrm{out}}+C_{\mathrm{CO}}^{\mathrm{out}}}\;\cdot 100$$

(4) $$Y_{{\mathrm{CH}}_{4}}\;(\%)\;=\frac{X_{{\mathrm{CO}}_{2}}{\cdot \;S}_{{\mathrm{CH}}_{4}}}{100}$$

where $C^{\mathrm{out}}$ is the molar concentration at the reactor outlet for each gas.

Lastly, Equation (5) was used to calculate the consumption rate of CO_2_ (mol g_cat_^−1^ s^−1^):

(5) $$r_{CO_{2}}=(\frac{X_{{\mathrm{CO}}_{2}}}{100})\cdot \left(\frac{F_{{\mathrm{CO}}_{2}}}{W_{\mathrm{cat}}}\right)$$

where $X_{CO_{2}}$ is the conversion of CO_2_ (%), $F_{CO_{2}}$ is the molar flow rate of CO_2_ entering the reactor (mol s^-1^), and $W_{cat}$ is the catalyst mass (g).

## 3. Results and Discussion

### 3.1. Characterization of the Supports and the Ni Catalysts

**Figure 1:**
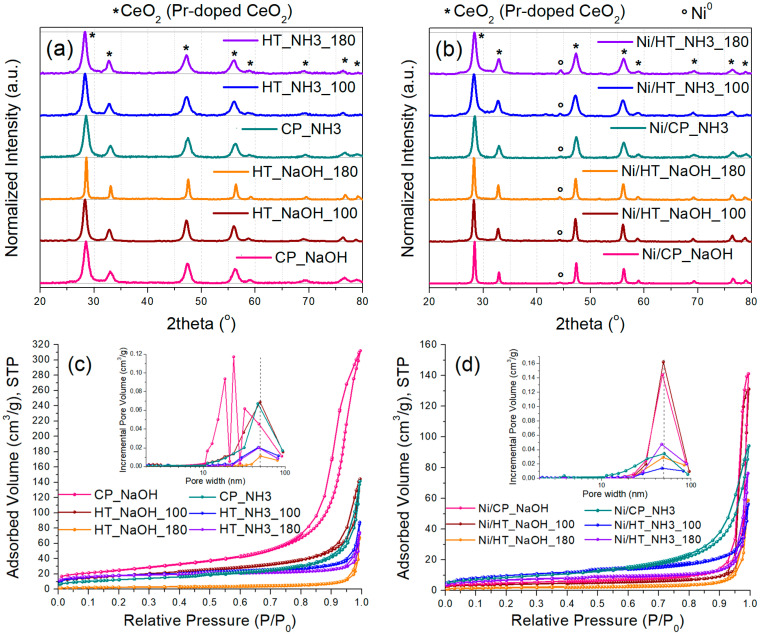
X-ray diffractograms of (**a**) the metal oxide supports and (**b**) the reduced Ni-supported catalysts. N_2_ physisorption isotherms along with pore size distribution (inset) of (**c**) the metal oxide supports and (**d**) the reduced Ni-supported catalysts.

At first, the prepared Pr-doped CeO_2_ metal oxide supports, and the corresponding Ni-supported catalysts, were characterized via XRD (Figure 1a,b). The Pr-doped CeO_2_ supports (Figure 1a) present the typical diffractions of the fluorite CeO_2_ lattice with a different peak broadening [18,20]. The calculated average crystallite sizes via the Scherrer equation fall in the range between 8 and 10 nm (Table 2), except for the HT_NaOH_180 support, which had an average crystallite size of around 20 nm. Therefore, the high basicity of the precipitating agent coupled with a high temperature for the hydrothermal treatment (180 °C) appears to favor the lattice growth of the Pr-doped CeO_2_ nanocrystallites. It is noted, that Pr is expected to readily dissolve into the CeO_2_ lattice of the support, as Pr^3+^ cations substitute Ce^4+^ ones, thereby causing lattice expansion when compared to undoped CeO_2_ [20,31].

The reduced Ni-supported catalysts were characterized next (Figure 1b). Besides the diffractions for the Pr-doped CeO_2_ support, diffractions attributed to metallic Ni^0^ are now also evident due to the presence of the supported Ni nanoparticles [20]. The calculated crystallite sizes for metallic Ni are between 8 and 15 nm (Table 2). The lowest crystallite size was calculated for Ni/HT_NaOH_100 (8 nm) and the highest for Ni/HT_NaOH_180 and Ni/CP_NaOH (15 and 14 nm, respectively). The catalysts whose support was prepared with mildly basic NH_3_-based buffer as the precipitating agent have a rather similar Ni crystallite size (11–13 nm), i.e., medium-sized Ni nanoparticles.

It is also interesting to note, that the diffraction broadening of the Pr-doped CeO_2_ crystallites changes in some cases from the supports to the reduced catalysts, namely for the Ni/HT_NaOH_100, and especially for the Ni/CP_NaOH catalysts. As a result, all the Ni-supported reduced catalysts prepared via NaOH as the precipitating agent display an almost double average crystallite size for Pr-doped CeO_2_ compared to those with NH_3_-based buffer as the precipitating agent. To elucidate the origin of this crystal growth in these cases, the calcined Ni-supported catalysts were also characterized (Figure S1), which display the crystalline diffractions for the support crystallites and the oxidized NiO particles. Since the peak broadening for Pr-doped CeO_2_ is similar between the bare supports and the calcined catalysts, it can be stated that the Pr-doped CeO_2_ crystallite growth in Ni/CP_NaOH and Ni/HT_NaOH_100 occurs during the following high-temperature (500 °C) reduction treatment under H_2_.

Afterwards, N_2_ physisorption isotherms were collected for the bare supports, as well as for the reduced Ni-supported catalysts (Figure 1c,d). The supports (Figure 1c) display a different porous structure for each material depending on the synthesis method. Most of them are mesoporous with a surface area ranging from 46 up to 93 m^2^/g (Table 2), with the highest surface area being recorded for the CP_NaOH sample, which also displays the highest pore volume. An outlier is HT_NaOH_180, which has much larger pore sizes and displays a quite low surface area of just 10 m^2^/g.

Regarding the reduced Ni-supported catalysts (Figure 1d), a general trend is that upon Ni impregnation/calcination followed by reduction, the pore volume and the surface area both drop, while the pore size distribution is shifted towards larger pore diameters. This is a result of the blocking of the smaller mesopores and pore reconstruction caused by the impregnation of Ni and the formation of supported metallic Ni nanoparticles [31,32]. For the cases of Ni/HT_NaOH_100 and especially Ni/CP_NaOH, a much higher extent of surface area loss occurs (even up to 80%), which is also consistent with the changes in the material crystallinity following Ni impregnation/calcination and reduction (i.e., significant growth of the Pr-doped CeO_2_ crystallites) observed during the XRD characterization. Overall, among all of the Ni-supported reduced catalysts, higher porosity is observed for those whose supports were prepared via the mildly basic NH_3_-based buffer as the precipitating agent.

**Figure 2:**
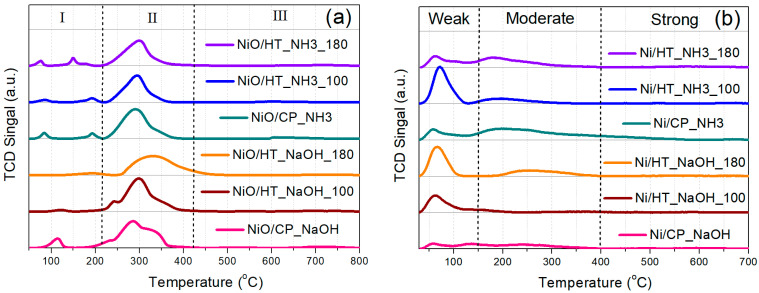
(**a**) H_2_-TPR profiles of the calcined catalysts. (**b**) CO_2_-TPD profiles of the reduced catalysts.

The catalyst material reducibility was investigated through H_2_-TPR on the calcined Ni-supported catalysts (Figure 2a). At the first region (I), below 200 °C, the observed reduction peaks could be ascribed to the reduction of highly dispersed NiO species at the catalyst surface, as well as possibly to Ni(OH)_2_ species, and Ni^2+^ solubilized in the Pr-doped CeO_2_ support [31,33]. At the second region (II), up to approx. 400 °C, the main (highest intensity) reduction peak can be assigned to the majority of NiO reduction to metallic Ni^0^, as well as to the contribution of the removal of surface oxygen species from the Pr-doped CeO_2_ support [31,33,34]. The peak of the main NiO reduction event is located between 290 and 300 °C for all materials, except for NiO/HT_NaOH_180, whose NiO reduction peak has the maximum at approx. 330 °C. This temperature range of NiO reduction to metallic Ni^0^ can be attributed to the high reducibility of NiO that is in contact with the defect-rich Pr-doped CeO_2_ support surface [31,35]. Lastly, the much smaller and quite broad peaks at higher reduction temperatures (region III) can be ascribed to oxygen removal from the bulk of the metal oxide support [31,33,34].

The surface basicity was then evaluated via CO_2_-TPD for the reduced Ni-supported catalysts (Figure 2b), which show three types of desorption peaks. The peaks at lower temperatures can be assigned to weakly bound carbonates and bicarbonates on the weak basic sites of the materials. The CO_2_ desorption peaks at the intermediate temperature range (approx. between 150 and 400 °C) are ascribed to carbonates that are formed over the moderately strong basic sites, whereas the small and broad peaks at higher temperatures are due to the strong basic sites [31,36,37]. Although some materials present quite intense CO_2_ desorption peaks at the low temperature range for the weak basic sites (e.g., Ni/HT_NH3_100), Ni/CP_NH3 is found to contain the highest amount of moderately strong basic sites at intermediate desorption temperatures. This can also be observed in Table S1, which includes the populations of weak, moderately strong, and strong basic sites for all catalysts. Ni/CP_NH3 contains the highest population of moderately strong basic sites, whereas the materials prepared with the NH_3_-based buffer have, in general, higher total basicity (including of moderate strength) compared to those prepared via NaOH. Based on the relevant literature [36,38,39], a higher population of moderately strong basic sites can be associated with a higher catalytic activity during CO_2_ methanation, since they act to enhance the CO_2_ chemisorption and activation during the catalytic reaction.

H_2_-TPD was carried out for the reduced Ni-supported catalysts (Figure S2). A first major hydrogen desorption peak arises in all samples at temperatures lower than 200 °C due to the weak binding of hydrogen atoms on the metallic Ni surface sites, whereas a smaller peak at higher temperatures is assigned to a stronger Ni-H binding [31,40]. The higher temperature peak is especially large for Ni/CP_NH3, and thus, the Ni-H binding appears to be stronger for this sample. The amounts of desorbed H_2_, as well as the calculated values for the Ni dispersion and the average Ni nanoparticle size are displayed in Table S2. In general, the Ni nanoparticle size values are larger compared to the average Ni crystallite sizes calculated via XRD (Table 2), possibly due to partial covering of the Ni nanoparticle surface with CeO_2_, thereby limiting H_2_ chemisorption [20,31]. Based on these calculations however, Ni/CP_NH3 appears to have the highest Ni dispersion among all the other catalysts.

Additionally, Raman characterization was performed for the reduced catalysts (Figure S3). The Raman spectra generally show a large peak at approx. 470 cm^−1^, which is the typical F_2G_ peak observed for CeO_2_-based materials with a CeO_8_ coordination [31,37,41]. The adjacent broad peak at higher wavenumbers is the so-called “defects” peak, which, for our materials with a Pr-doped CeO_2_ support structure, can be separated into two types of contributions, namely one originating from mainly extrinsic oxygen vacancies (O_V_) at 540 cm^–1^ due to aliovalent (Pr^3+^) doping of the CeO_2_ support, and one originating from the presence of some amount of PrO_x_ heterophase (PrO_8_ coordination) in the range of 600–630 cm^–1^ [18,20,41,42]. The I_Ov_/I_F2G_ ratio values, indicative of the oxygen vacancy population or rather the relative abundance of oxygen vacancies, are summarized in Table S3. The highest O_V_ contribution is observed for the Ni/HT_NaOH_100, Ni/CP_NaOH, and Ni/CP_NH3 catalysts. It should be noted herein, that a high oxygen vacancy population can benefit the CO_2_ methanation catalytic performance, since they can act as CO_2_ adsorption sites, as well as enhance the oxygen mobility of the support and enable a faster conversion of the reaction intermediates [14,15,33,43].

**Figure 3:**
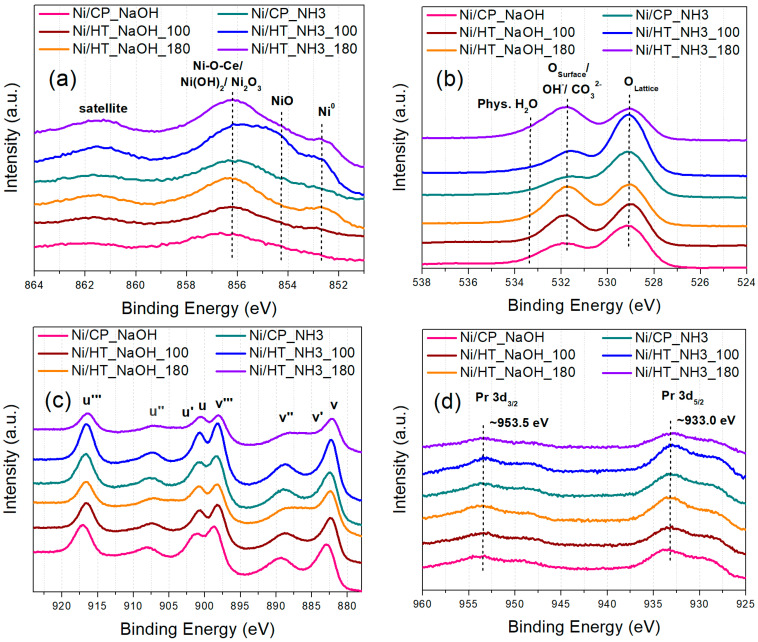
(**a**) Ni2p, (**b**) O1s, (**c**) Ce3d, and (**d**) Pr3d XPS core level spectra for the reduced catalysts.

The catalysts’ surface chemistry was then studied via XPS, which was conducted ex situ (Figure 3). The Ni2p spectra (Figure 3a) reveal the following types of surface Ni-species, with increasing binding energy (BE): (i) metallic Ni^0^ surface sites at lower BE, (ii) then NiO surface sites which originate following ex situ oxidation of formerly metallic surface Ni sites, and, (iii) finally, at higher BE, the large peak can be ascribed to the contribution of Ni(OH)_2_, which can arise following surface Ni oxidation and hydroxylation, Ni_2_O_3_ or Ni^3+^ due to defects in the NiO structure, and Ni species at the Ni-CeO_2_ interface (Ni-O-Ce sites) [20,34,44]. Regarding the O1s spectra (Figure 3b), these can be separated into surface oxygen from the metal oxide support lattice (Pr-doped CeO_2_) at lower BE, and adsorbed oxygen species, like hydroxyls and carbonates on the catalyst surface, and also physisorbed H_2_O, at higher BE [20,45]. The majority of these adsorbed oxygen species at higher BE are also expected to originate following atmospheric exposure [20]. Although differences in the peak shapes are observed for the different catalysts, these can be rather attributed to the exposure of the samples to atmospheric oxygen, and to a different extent of surface oxidation and hydroxylation. In particular, the entirety of the Ni phase is expected to be initially metallic following the reduction treatment at 500 °C, as was verified during H_2_-TPR characterization (Figure 2a), and thus, the oxidized Ni species in the materials originate during the subsequent atmospheric exposure.

The Ce3d spectra (Figure 3c) show the presence of multiple peaks due to the Ce3d_5/2_ (peaks labeled as v) and Ce3d_3/2_ (peaks labeled as u) transitions. Since Ce ions typically exist in both Ce^4+^ and Ce^3+^ oxidation states in CeO_2_-based oxides, the peaks v, v″, v‴, u, u″, and u‴ can be ascribed to the major Ce^4+^ oxidation state, whereas the peaks v′ and u′ correspond to the minority Ce^3+^ ions due to intrinsic defects in the oxide support lattice [18,20]. However, the majority of the oxygen vacancy sites are expected to originate via the extrinsic substitutional defects of aliovalent Pr^3+^ ions in former Ce^4+^ sites [20,46]. In the Pr3d spectra (Figure 3d), the position and peak shape of the Pr3d_5/2_ and Pr3d_3/2_ transition peaks resemble those of a Pr_2_O_3_-like oxide, thus confirming that Pr rather exists in the Pr^3+^ oxidation state [20,47]. These extrinsic (Pr_Ce_′) substitutional defects can generate a large number of oxygen vacancy sites, which can significantly promote the CO_2_ methanation catalytic activity [14,15,33].

The elemental surface concentrations measured via XPS can be found in Table S4. The differences in adventitious carbon and the surface concentration of some elements can be attributed to atmospheric exposure, although they roughly agree with the expected values. A more pronounced Ni concentration at the surface is expected, since Ni is mainly located as surface metallic nanoparticles, whereas the higher Pr surface concentration can be attributed to a certain extent of PrO_x_ segregation at the support grains [20,48]. An interesting finding is that a significant Na surface concentration was detected for the reduced catalysts whose supports were prepared using NaOH as the precipitating agent (also observed via the intensity of the Na1s XPS peaks in Figure S4), even though the typical washing steps were applied during the material preparation procedures to remove these ions, similarly to other works [22,25,26,49,50,51]. This can be important during the subsequent catalytic evaluation of the materials, since Na can potentially act as a catalyst inhibitor (through electronic modifications by inducing a positive charge on Ni, and by blocking active surface sites) and has often been reported to impair the CO_2_ methanation catalytic performance [52,53,54,55,56].

Additionally, peak deconvolutions for the XPS core level spectra of Ni2p, O1s, and Ce3d were conducted, and the deconvoluted peaks are shown in Figures S5–S7. Furthermore, the calculated percentages of metallic Ni (Ni^0^), adsorbed oxygen species (O_Ads_) in relation to lattice oxygen species (O_lat_), and Ce^3+^ species are shown in Table S5. As mentioned before, the low percentage of metallic Ni^0^ can be attributed to the prior air exposure of the samples, and, according to prior works [20,31], this phenomenon can be exacerbated for smaller Ni particles. There are various possible nickel oxide/hydroxide species having similar binding energies (in the 853–857 eV region) and complex peak shapes, due to multiple splitting effects, particularly after air exposure [57]. As such, the quantified data from these peak fits (Table S5) should be treated with caution. The percentage of adsorbed oxygen species is also affected by air exposure (including the presence of adsorbed hydroxyl and carbonate species), but it can be observed however, that there are more adsorbed oxygen species for the samples that were hydrothermally treated under a higher temperature (180 °C, for Ni/HT_NaOH_180 and Ni/HT_NH3_180). These adsorbed oxygen species (hydroxyls and carbonates) can also form very thin surface layers, in addition to being physiochemically absorbed. Lastly, regarding the Ce^3+^ content, this also presents some variations, with the highest content observed for the samples hydrothermally treated at 180 °C. Nevertheless, as already mentioned before and in our prior works [20,31], for this type of Pr-doped CeO_2_ supports, the majority of the oxygen vacancies originate via Pr-doping, i.e., via Pr_Ce_′ defects (the Pr^3+^ state is also verified via the Pr3d XPS spectra). Positively charged oxygen vacancies are readily generated close to these Pr_Ce_′ substitutional defects for charge compensation, and this process can leave the nearby Ce-cations in their higher oxidation state (Ce^4+^) [20].

**Figure 4:**
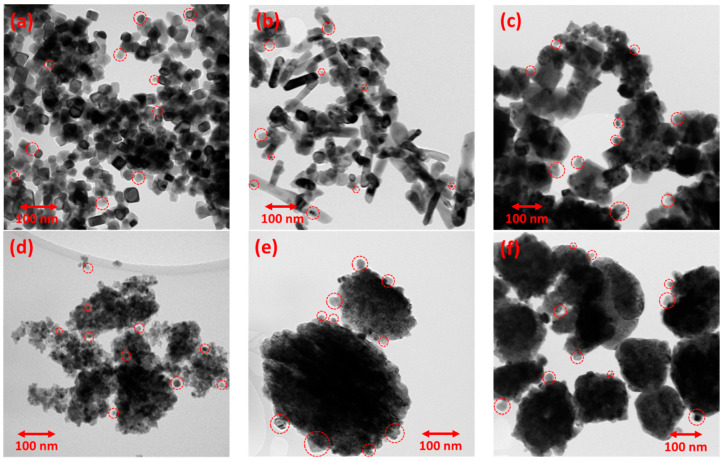
TEM images of the (**a**) Ni/CP_NaOH, (**b**) Ni/HT_NaOH_100, (**c**) Ni/HT_NaOH_180, (**d**) Ni/CP_NH3, (**e**) Ni/HT_NH3_100, and (**f**) Ni/HT_NH3_180 reduced Ni-supported catalysts. The red circles indicate the location of some Ni nanoparticles.

Lastly, electron microscopy analysis was performed to determine the material nanostructure. TEM images of the reduced Ni-supported catalysts are displayed in Figure 4, whereas the corresponding images of the bare Pr-doped CeO_2_ metal oxide supports can be found in Figure S8. The reduced catalysts are comprised of the metal oxide support nanostructure alongside the supported spherical-shaped metallic Ni nanoparticles. The majority of the supported Ni nanoparticles are approx. 5–20 nm in diameter (medium-sized), although some of them are somewhat larger, even up to 50 nm. An accurate estimation of the Ni nanoparticle size via TEM is challenging, due to the low Z-contrast in the TEM images. Nevertheless, Ni nanoparticle size distribution histograms for all six catalysts were constructed and can be found in Figure S9, but, as already mentioned, these data should be treated with caution. Interestingly, however, the lowest average Ni nanoparticle size was calculated for Ni/CP_NH3, which agrees with the H_2_-TPD results.

From TEM, it is apparent that the metal oxide support nanostructure changes depending on the synthesis method used to prepare it. Regarding the materials prepared using highly basic NaOH as the precipitating agent, the one with no hydrothermal treatment (i.e., co-precipitation, Ni/CP_NaOH, Figure 4a) presents small and rather cubic nanoparticles for the metal oxide support, whereas the ones with the hydrothermal treatment at 100 and 180 °C (Ni/HT_NaOH_100 and Ni/HT_NaOH_180, Figure 4b,c) present the typical nanostructures of nanorods and nanocubes, respectively, due to the preferred crystal growth along particular crystalline facets, as also observed in the other literature works [21,22,24,25,50]. For the materials prepared using the mildly basic NH_3_-based buffer as the precipitating agent, the one with no hydrothermal treatment (i.e., co-precipitation, Ni/CP_NH3, Figure 4d) presents a rather unstructured mesoporous morphology consisting of aggregates of small crystallites, while the ones with the hydrothermal treatment at 100 and 180 °C (Ni/HT_NH3_100 and Ni/HT_NH3_180, Figure 4e,f) reveal the formation of large particle aggregates for the metal oxide support with a diameter higher than 100 nm, but these also consist of smaller aggregated crystallites.

The TEM images of the bare metal oxide supports, i.e., prior to Ni impregnation/calcination and reduction, are shown in Figure S8. Nanorods are observed for HT_NaOH_100, nanocubes for HT_NaOH_180, unstructured mesoporous morphology of aggregated crystallites for CP_NH3, and large aggregated particles for HT_NH3_100 and HT_NH3_180. The notable exception is CP_NaOH, which presents a quite different morphology for the metal oxide support compared to Ni/CP_NaOH. As mentioned during the XRD characterization results, this change/consolidation in the nanostructure to small cubic nanoparticles followed by crystal growth for Pr-doped CeO_2_ occurs during the high-temperature reduction treatment after the Ni impregnation and calcination.

**Figure 5:**
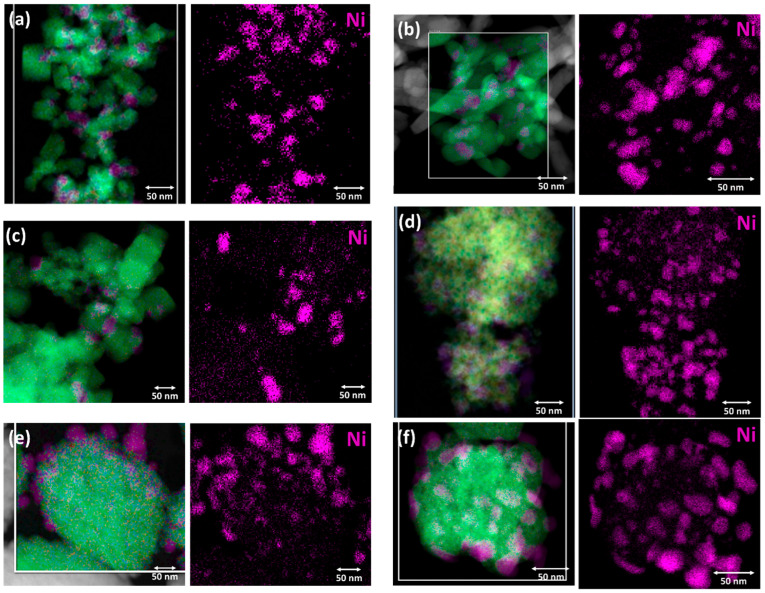
HAADF-STEM EDS elemental mapping images and EDS elemental mapping for Ni for the (**a**) Ni/CP_NaOH, (**b**) Ni/HT_NaOH_100, (**c**) Ni/HT_NaOH_180, (**d**) Ni/CP_NH3, (**e**) Ni/HT_NH3_100, and (**f**) Ni/HT_NH3_180 reduced Ni-supported catalysts.

A more precise localization of the metallic Ni supported phase, as well as the determination of the elemental distribution across the materials, is achieved via HAADF-STEM and EDS elemental mapping (Figure 5 and Figure S10). Figure 5 shows the Ni nanoparticle distribution across the catalyst materials, with most of them being medium-sized (5–20 nm in diameter), whereas some larger Ni nanoparticles even up to 50 nm in diameter can also be observed. Medium-sized Ni nanoparticles supported over CeO_2_-based oxides have been previously reported to be quite efficient during CO_2_ methanation [31,51,58]. The Ni nanoparticles are located between the cubic support grains, nanorods, and nanocubes for the materials prepared with NaOH as the precipitating agent, throughout the unstructured mesoporous support morphology for Ni/CP_NH3, and, for Ni/HT_NH3_100 and Ni/HT_NH3_180, they appear to preferentially reside at the outer surface of the large support particle aggregates. The other elements (O, Ce, and Pr) are found to be evenly distributed across the supports, thereby also verifying that Pr (as Pr^3+^) is solubilized into the CeO_2_ crystalline lattice. An example of the entire elemental distribution (O, Ce, Pr, and Ni) for Ni/CP_NH3 is shown in Figure S10. Moreover, in agreement with the XPS results (Table S4), Na was also observed in the three catalysts prepared with NaOH. The Na EDS mapping images for Ni/CP_NaOH, Ni/HT_NaOH_100, and Ni/HT_NaOH_180 can be found in Figure S11.

Even though the magnification during TEM characterization is not very large, some conclusions on the exposed CeO_2_ crystalline facets for the supports (Pr-doped CeO_2_) can be derived, also based on the relevant literature. For the samples prepared with the NH_3_-based buffer, the individual CeO_2_ nanocrystals cannot be distinguished easily. However, by taking into account the work of Bian et al. [25], we could assume a preferential exposure of the (111) CeO_2_ facet for small CeO_2_ nanoparticles, in line with their respective materials. Regarding the materials with well-defined nanorod and nanocube support morphologies, based on the thorough characterizations performed by Bian et al. [25] and Hashimoto et al. [21], the CeO_2_ nanocubes present (100) exposed facets, and the CeO_2_ nanorods both (100) and (110) exposed facets. For Ni/CP_NaOH, we observe CeO_2_ nanocubes; however, these are quite small, and other facets can also be exposed.

### 3.2. Catalytic Activity

**Figure 6:**
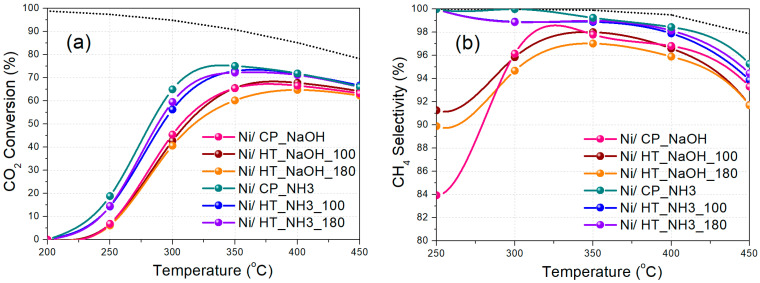
(**a**) CO_2_ conversion and (**b**) CH_4_ selectivity as a function of reaction temperature (Experimental Protocol #1). The thermodynamic equilibrium (dotted lines) is calculated via Aspen Plus (*p* = 1 atm and H_2_:CO_2_ = 4:1).

The CO_2_ methanation catalytic activity was then evaluated as a function of reaction temperature at two different WHSV values, namely at 25,000 mL g_cat_^−1^ h^−1^ for Experimental Protocol #1, and at 100,000 mL g_cat_^−1^ h^−1^ for Experimental Protocol #2. During Experimental Protocol #1 (Figure 6), a clear observation regarding the catalytic activity can be made, that the materials prepared using mildly basic NH_3_-based buffer as the precipitating agent are significantly more active when compared to the materials prepared using highly basic NaOH as the precipitating agent, with the former being able to reach much higher CO_2_ conversion and CH_4_ selectivity values at lower reaction temperatures. Repeat experiments were performed to demonstrate result reproducibility, and the corresponding error bars for Ni/CP_NH3 can be found in Figure S12. In all cases, the relative standard deviation was calculated at below 5%. Additionally, the corresponding CH_4_ yield values for the experiments are shown in Figure S13a.

Regarding the materials prepared with NaOH as the precipitating agent, the one with no hydrothermal treatment (Ni/CP_NaOH) and the one with the hydrothermal treatment at 100 °C (Ni/HT_NaOH_100) display a similar catalytic activity, whereas the one with the hydrothermal treatment at 180 °C (Ni/HT_NaOH_180) presents a lower one. This is in agreement with other literature works reporting that Ni nanoparticles supported on CeO_2_ with nanorod morphology are more active than those supported on CeO_2_ with nanocube morphology, which is typically assigned to differences in the oxygen reactivity of the respective exposed crystalline facets (e.g., (110) vs. (100) CeO_2_ facets) [21,22,23,24]. Nevertheless, the significantly inferior catalytic activity of all of these three catalysts compared to those prepared with the NH_3_-based buffer as the precipitating agent can be attributed to: (i) the lower surface area accompanied by a larger Pr-doped CeO_2_ crystallite size, and (ii) the lower basic site population, including for the moderately-strong basic sites [31,38]. The significant presence of residual Na on the catalyst surface (Table S4, Figure S11), despite the washing treatments during catalyst preparation, is most probably also responsible for the lower CO_2_ conversion and CH_4_ selectivity values, in agreement with the other relevant literature works [52,53,54,55].

Regarding the materials prepared with the NH_3_-based buffer as the precipitating agent, the ones prepared following hydrothermal treatment at 100 °C (Ni/HT_NH3_100) and 180 °C (Ni/HT_NH3_180), leading to large particle aggregates for the metal oxide support, have a rather similar catalytic activity between them. Overall, however, the best catalytic performance, especially at the low-temperature regime, is obtained for the Ni/CP_NH3 catalyst prepared via co-precipitation, leading to a maximum CO_2_ conversion of 75% (with 99% CH_4_ selectivity) at 350 °C (Table 3). The CO_2_ conversion and CH_4_ selectivity values then drop at higher temperatures, due to the exothermicity of CO_2_ methanation and the promotion of the antagonistic reverse water–gas shift reaction [9].

It is thus found herein, that a rather simple catalyst support (Pr-doped CeO_2_) preparation procedure, with an NH_3_-based buffer as the precipitating agent and in the absence of hydrothermal treatment, yielding an unstructured mesoporous morphology for the support, leads to an eventually higher CO_2_ methanation catalytic activity when compared to the materials prepared using highly basic NaOH and hydrothermal treatments that yield specific, well-defined catalyst support nanostructures such as nanorods and nanocubes. This can be ascribed to the high specific surface area (Table 2, N_2_ physisorption), high population of basic sites, particularly of moderate strength (Figure 2b/Table S1, CO_2_-TPD), favorable Ni dispersion (Table S2, H_2_-TPD) and defect chemistry/oxygen vacancy population (Figure S3/Table S3, Raman), as well as the absence of catalyst inhibitors such as residual Na [16,31,38,54]. It could also be assumed, that the (111) CeO_2_ facet exposure in small Pr-doped CeO_2_ nanoparticles (prepared with the NH_3_-based buffer) can promote the CO_2_ methanation catalytic performance, according to Bian et al. [25].

The significantly lower catalytic performance of all the materials prepared with NaOH, compared to those prepared with the NH_3_-based buffer, experimentally verifies the inhibitory effect of Na that is thoroughly discussed in other works (electronic modification and surface coverage of the active Ni metallic phase) [53,54,55,56]. An Le et al. [53] observed an inhibitory effect of Na on CO_2_ methanation over Ni/CeO_2_, even at 0.1 wt%. Wu et al. [54] observed a similar effect of Na for Ni/SiO_2_, in addition to a higher CO selectivity over CH_4_, and attributed this to the presence of a highly positive charge over the Ni metal upon Na introduction, and the weaker binding of CO and H_2_ due to the blocking of active metal surface sites. Furthermore, Beierlein et al. [55] found that, for Ni/Al_2_O_3_, the presence of leftover Na following deposition precipitation and co-precipitation synthesis increases the CO selectivity over CH_4_, whereas Chen et al. [56] concluded that NiNa_x_/Al_2_O_3_ catalysts can be quite effective to produce carbon nanofibers and CO during CO_2_ hydrogenation, to the detriment of CH_4_ production. Additionally, the fact that the best performance was obtained without the hydrothermal treatment (for Ni/CP_NH3) negates the significance of this method as a means to achieve high catalytic activity.

Therefore, by taking into account that the best catalytic performance was obtained via co-precipitation synthesis and mildly basic NH_3_-based buffer, the potential for industrial applicability for the best-performing catalyst (Ni/CP_NH3) is enhanced, since the energy needs for the hydrothermal treatment, and the need for high-pH wastewater management (generated via NaOH-based synthesis), can be avoided. In general, among all the different catalysts, the best-performing one, Ni/CP_NH3, would have the lowest production costs on an industrial scale.

**Figure 7:**
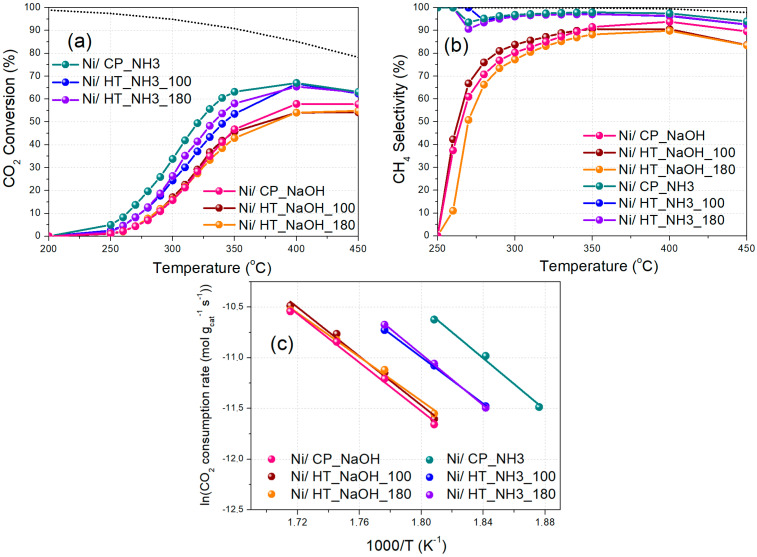
(**a**) CO_2_ conversion and (**b**) CH_4_ selectivity as a function of reaction temperature (Experimental Protocol #2). (**c**) Arrhenius plots (logarithm of the CO_2_ consumption rate vs. 1000/T). The thermodynamic equilibrium (dotted lines) is calculated via Aspen Plus (*p* = 1 atm and H_2_:CO_2_ = 4:1).

The catalysts were then evaluated at a higher WHSV of 100,000 mL g_cat_^−1^ h^−1^ and with additional temperature steps during Experimental Protocol #2 (Figure 7). The increase in the total flow to catalysts weight (F/W) ratio leads to lower CO_2_ conversion and CH_4_ selectivity values compared to the results obtained via Experimental Protocol #1 (higher F/W ratio and lower WHSV) [31,58]. Again, it is found that Ni/CP_NH3 displays the best catalytic performance, with higher CO_2_ conversion and CH_4_ selectivity values, especially at the low-temperature regime, followed by Ni/HT_NH3_180 and Ni/HT_NH3_100. On the other hand, the catalysts prepared with NaOH as the precipitating agent display a substantially inferior CO_2_ methanation catalytic performance. The corresponding CH_4_ yield values are shown in Figure S13b. The measurements taken for CO_2_ conversion values below 20%, and thus at the kinetically controlled regime, allow for the calculation of the activation energy values via the Arrhenius plots (Figure 7c), assuming pseudo-first order reaction kinetics [31]. These values can be found in Table 3 and fall in the range between 90 and 110 kJ/mol, as would be expected for Ni/CeO_2_ type catalysts [22,38,59,60].

### 3.3. Catalytic Stability and Spent Catalyst Characterization

**Figure 8:**
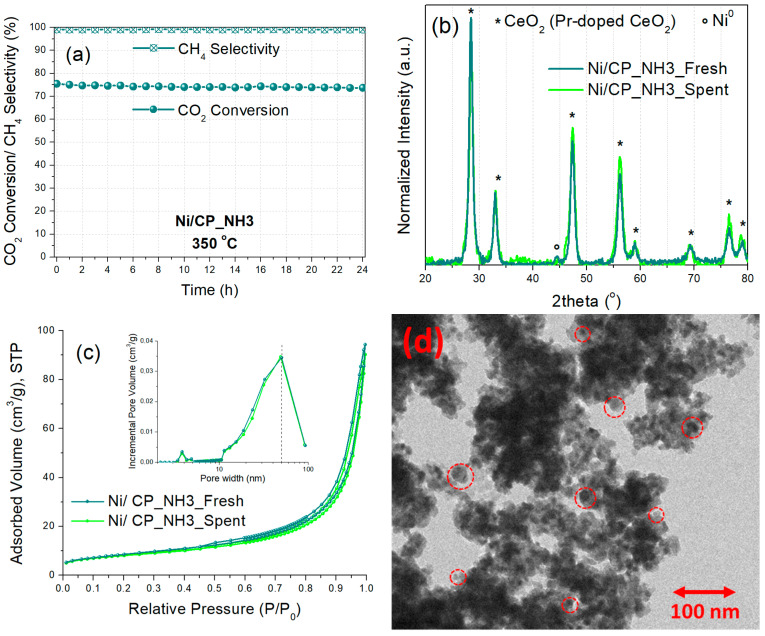
(**a**) Time-on-stream catalytic stability for Ni/CP_NH3 at 350 °C for 24 h (Experimental Protocol #3). (**b**) X-ray diffractograms and (**c**) N_2_ physisorption isotherms and pore size distribution graphs (inset) of the fresh (reduced) and spent Ni/CP_NH3 catalysts. (**d**) TEM image of spent Ni/CP_NH3. The red circles indicate the location of some Ni nanoparticles.

The stability of the best-performing Ni/CP_NH3 catalyst was then evaluated at a constant temperature of 350 °C and WHSV of 25,000 mL g_cat_^−1^ h^−1^, initially for a duration of 24 h, under Experimental Protocol #3 (Figure 8a). The CO_2_ conversion remained quite stable, with a minor drop to 74%, while the CH_4_ selectivity also remained constant at 99%. Moreover, in order to better demonstrate the catalytic stability under a higher time-on-stream duration, the spent catalyst was then reloaded in the reactor and tested for an additional 24 h (48 h time-on-stream in total, Figure S14). The CO_2_ conversion dropped to 73% (just 3% total drop), with the CH_4_ selectivity remaining at 99%. This high catalytic stability is also in line with other literature works studying similar catalysts [19,24,25,26]. It is thus shown, that Ni/CP_NH3 can provide a high and stable catalytic performance under long-term operation.

The spent Ni/CP_NH3 catalyst (after 24 h time-on-stream) was characterized via XRD, N_2_ physisorption, and TEM, to examine potential catalyst deactivation effects. The X-ray diffractograms (Figure 8b) of the fresh (reduced) and the spent catalyst largely overlap, showing no significant changes in the catalyst material crystallinity, although the average crystallite size of Ni is now calculated at 13 nm for the spent catalyst, compared to 11 nm for the fresh one. The N_2_ physisorption results (Figure 8c) also show similar textural properties for the spent catalyst, with a specific surface area of 29 m^2^/g, pore volume of 0.13 cm^3^/g, and average pore diameter of 19 nm, i.e., quite similar values to those obtained for the fresh catalyst (Table 2). Furthermore, TEM characterization of the spent Ni/CP_NH3 catalyst (Figure 8d) displays a similar unstructured mesoporous morphology of aggregated small crystallites for the metal oxide support, with medium-sized (5–20 nm) supported Ni nanoparticles. Therefore, it can be stated that Ni/CP_NH3 largely retains its crystallinity, nanomorphology, and Ni dispersion during the time-on-stream operation, with just a potentially minor extent of Ni nanoparticle sintering.

It should be noted herein, that the CO_2_ methanation catalytic performance has also been previously studied under the presence of gas impurities such as H_2_S, H_2_O, and O_2_. Dou et al. [61] and Méndez-Mateos et al. [62] found that La-doping could improve the catalyst resistance towards H_2_S poisoning, whereas Spataru et al. [63] found that the Ni catalysts supported on zeolites provided a higher resistance to H_2_O and O_2_ compared to the Al_2_O_3_-supported catalyst. As such, the testing of our best-performing catalyst (Ni/CP_NH3) under the presence of such gas impurities is an interesting topic for a further up-scaling study.

## 4. Conclusions

This work reports on the co-precipitation and hydrothermal synthesis of Pr-doped CeO_2_ support nanostructures for Ni/Pr-CeO_2_ CO_2_ methanation catalysts by varying the basicity of the precipitating solution and the hydrothermal treatment temperature, thus also investigating the potential advantages of the hydrothermal treatment and the use of a highly basic precipitating solution. It is found that different catalyst support nanostructures can be obtained depending on the support synthesis method, ranging from structured nanorods and nanocubes when using highly basic NaOH and elevated hydrothermal treatment temperature (100 and 180 °C, respectively) to an unstructured mesoporous support morphology consisting of aggregated small crystallites when employing a mildly basic NH_3_-based buffer as the precipitating solution in the absence of hydrothermal treatment. In all cases, medium-sized Ni nanoparticles are supported on the metal oxide support nanostructures.

Catalytic activity evaluation showed that the catalysts prepared with the mildly basic NH_3_-based buffer as the precipitating agent performed significantly better during CO_2_ methanation, with Ni/CP_NH3 synthesized via co-precipitation (instead of hydrothermal method) leading to the best results. On the contrary, the more structured catalyst support nanostructures prepared with highly basic NaOH led to inferior catalytic performance, due to the rather unfavorable physicochemical properties (lower surface area and basic site population, including of moderate strength), and also due to the catalyst inhibition by residual Na from the synthesis procedure. Furthermore, the best-performing Ni/CP_NH3 catalyst was shown to be highly stable with limited deactivation during time-on-stream operation.

In short, this study shows that the utilization of a rather simple catalyst support preparation procedure, i.e., co-precipitation in the absence of hydrothermal treatment and with the use of a mildly basic precipitating solution, can be more beneficial toward CO_2_ methanation when compared to the preparation of highly ordered catalyst support nanostructures that require highly basic precipitating solution and hydrothermal treatment.

## Figures and Tables

**Table 1:** Overview of the different precipitating agents and hydrothermal treatments used to prepare each Pr-doped CeO_2_ metal oxide support.

Catalyst Support	Precipitating Agent	Hydrothermal Treatment Temperature
CP_NaOH	NaOH	R.T. ^1^ (Co-precipitation)
HT_NaOH_100	NaOH	100 °C
HT_NaOH_180	NaOH	180 °C
CP_NH3	NH_3_/(NH_4_)_2_CO_3_	R.T. ^1^ (Co-precipitation)
HT_NH3_100	NH_3_/(NH_4_)_2_CO_3_	100 °C
HT_NH3_180	NH_3_/(NH_4_)_2_CO_3_	180 °C

^1^ R.T. = Room temperature.

**Table 2:** Crystallite sizes of CeO_2_ (Pr-doped CeO_2_, Φ_CeO2_) and Ni^0^ (Φ_Ni_) calculated from XRD through the Scherrer equation. Specific surface area (SSA), pore volume (V_P_), and average pore diameter (D_ave_) determined via N_2_ physisorption. Values are provided for both the Pr-doped CeO_2_ supports and the corresponding Ni-supported reduced catalysts.

Support/Catalyst	$\Phi_{CeO_{2}}$ (nm)	$\Phi_{Ni^{0}}$ (nm)	SSA (m^2^/g)	$V_{P}$ (cm^3^/g)	$D_{ave}\;(\mathrm{nm})$
CP_NaOH	8	n.a.	91	0.48	21
HT_NaOH_100	10	n.a.	63	0.22	14
HT_NaOH_180	20	n.a.	10	0.10	38
CP_NH3	8	n.a.	46	0.21	18
HT_NH3_100	9	n.a.	61	0.12	8
HT_NH3_180	9	n.a.	58	0.11	7
Ni/CP_NaOH	22	15	17	0.22	50
Ni/HT_NaOH_100	17	8	13	0.20	61
Ni/HT_NaOH_180	20	14	8	0.09	48
Ni/CP_NH3	11	11	31	0.14	19
Ni/HT_NH3_100	9	13	35	0.08	10
Ni/HT_NH3_180	10	13	24	0.12	19

**Table 3:** CO_2_ methanation catalytic performance metrics at 350 °C, and in parenthesis at 300 °C, calculated via Experimental Protocol #1. Activation energy values calculated via Experimental Protocol #2.

Catalyst	CO_2_ Conversion (%)	CH_4_ Selectivity (%)	CH_4_ Yield (%)	Activation Energy (kJ/mol)
Ni/CP_NaOH	65 (45)	98 (96)	64 (44)	101
Ni/HT_NaOH_100	66 (43)	98 (96)	64 (41)	100
Ni/HT_NaOH_180	60 (41)	97 (95)	58 (38)	91
Ni/CP_NH3	75 (65)	99 (100)	75 (65)	106
Ni/HT_NH3_100	73 (56)	99 (99)	72 (56)	95
Ni/HT_NH3_180	72 (59)	99 (99)	72 (59)	104

## Data Availability

Dataset available on request from the authors.

## Supplementary Materials

The following supporting information can be downloaded at: https://www.mdpi.com/article/10.3390/nano15131022/s1, Figure S1: X-ray diffractograms of the calcined catalysts; Table S1: Populations of weak, moderately strong, and strong basic sites; Figure S2: H_2_-TPD profiles; Table S2: Data derived from H_2_-TPD, including Ni dispersion; Figure S3: Raman spectra; Table S3: I_OV_/I_F2G_ ratios from Raman analysis; Table S4: XPS elemental surface concentrations; Figure S4: Na1s XPS core level spectra; Figure S5: Ni2p XPS peak deconvolution; Figure S6: O1s XPS peak deconvolution; Figure S7: Ce3d XPS peak deconvolution; Table S5: Data derived from XPS peak deconvolutions; Figure S8: TEM images of the calcined metal oxide supports; Figure S9: Ni nanoparticle size distribution histograms; Figure S10: HAADF-STEM along with EDS elemental mapping for Ni/CP_NH3; Figure S11: Na EDS mapping images for Ni/CP_NaOH, Ni_HT_NaOH_100, and Ni/HT_NaOH_180; Figure S12: Average values and error bars from 3 catalytic experiments performed for Ni/CP_NH3; Figure S13: CH_4_ yield as a function of reaction temperature; Figure S14: Time-on-stream catalytic stability for a total of 48 h for Ni/CP_NH3.
