# Supplementary material for: CO2 Methanation over Ni Catalysts Supported on Pr-Doped CeO2 Nanostructures Synthesized via Hydrothermal and Co-Precipitation Methods
Source: Nanomaterials (Basel). 2025 Jul 1;15(13):1022. doi: 10.3390/nano15131022 (PMC12250989; doi:10.3390/nano15131022)
Supplement: Supplementary file 1 [file nanomaterials-15-01022-s001.zip › nanomaterials-3684882-supplementary.pdf]

CO_2 methanation over Ni catalysts supported on Pr-doped CeO_2 nanostructures synthesized via hydrothermal and co-precipitation methods

Anastasios I. Tsiotsias[1], Nikolaos D. Charisiou[1,*], Aasif A. Dabbawala[2,3], Aseel G.S. Hussien[2,3], Victor Sebastian[4,5,6], Steven J. Hinder[7], Mark A. Baker[7], Samuel Mao[2], Kyriaki Polychronopoulou[2,3] and Maria A. Goula[1,8,9,*]

[1]Laboratory of Alternative Fuels and Environmental Catalysis (LAFEC), Department of Chemical Engineering, University of Western Macedonia, GR-50100, Kozani, Greece

[2]Department of Mechanical Engineering, Khalifa University of Science and Technology, Abu Dhabi, P.O. Box 127788, UAE

[3]Center for Catalysis and Separations, Khalifa University of Science and Technology, Abu Dhabi, P.O. Box 127788, UAE

[4]Department of Chemical Engineering and Environmental Technology, Universidad de Zaragoza, Campus Río Ebro-Edificio I+D, 50018 Zaragoza, Spain

[5]Instituto de Nanociencia y Materiales de Aragón (INMA), Universidad de Zaragoza- CSIC, c/ María de Luna 3, 50018 Zaragoza, Spain

[6]Networking Research Center on Bioengineering, Biomaterials and Nanomedicine, CIBERBBN, 28029 Madrid, Spain

[7]The Surface Analysis Laboratory, Faculty of Engineering and Physical Sciences, University of Surrey, Guildford, GU2 4DL, UK

[8]Centre for Research & Technology Hellas (CERTH), Chemical Process and Energy Resources Institute (CPERI), 52 Egialias Str., Athens 15125, Greece

[9]School of Science and Technology, Hellenic Open University, Parodos Aristotelous 18, Patras 26335, Greece

*Corresponding authors:

Asst. Prof. Nikolaos D. Charisiou (ncharisiou@uowm.gr)
University of Western Macedonia, Department of Chemical Engineering, Laboratory of Alternative Fuels and Environmental Catalysis, ZEP, Kozani, 50100, Greece, Telephone: +302461056551

Prof. Maria A. Goula (mgoula@uowm.gr)
University of Western Macedonia, Department of Chemical Engineering, Laboratory of Alternative Fuels and Environmental Catalysis, ZEP, Kozani, 50100, Greece, Telephone: +302461056551

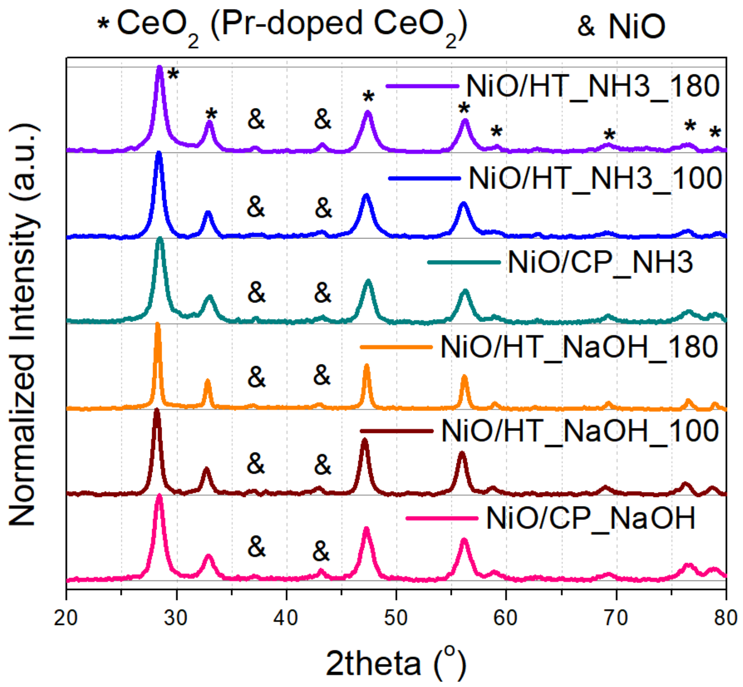

Figure S1. X-ray diffractograms of the calcined Ni-supported catalysts.

Table S1. Populations of weak, moderately strong, and strong basic sites (μmol/g) on the reduced catalysts, calculated from the CO_2-TPD profiles.

Catalyst	Weak (<150 °C, μmol/g)	Moderately strong (150 - 400 °C, μmol/g)	Strong (>400 °C, μmol/g)	Total (μmol/g)
Ni/CP_NaOH	11	12	1	24
Ni/HT_NaOH_100	51	9	3	63
Ni/HT_NaOH_180	54	21	2	77
Ni/CP_NH3	34	54	7	95
Ni/HT_NH3_100	77	19	3	99
Ni/HT_NH3_180	37	41	4	82

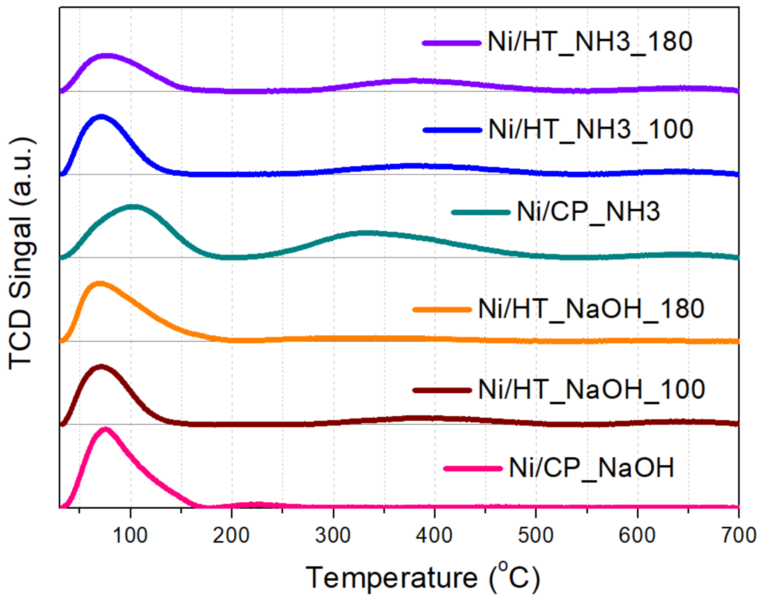

Figure S2. H_2 TPD profiles of the reduced catalysts.

Table S2. Amount of H_2 desorbed during H_2-TPD (μmol/g), Ni dispersion (%), and average Ni nanoparticle size (nm) calculated via H_2-TPD.

Catalyst	H_2 desorbed (μmol/g)	Ni dispersion (%)	Average Ni nanoparticle size (nm)
Ni/CP_NaOH	46	5.5	18
Ni/HT_NaOH_100	39	4.7	21
Ni/HT_NaOH_180	42	5.1	19
Ni_CP_NH3	63	7.6	13
Ni/HT_NH3_100	42	5.1	19
Ni/HT_NH3_180	37	4.5	22

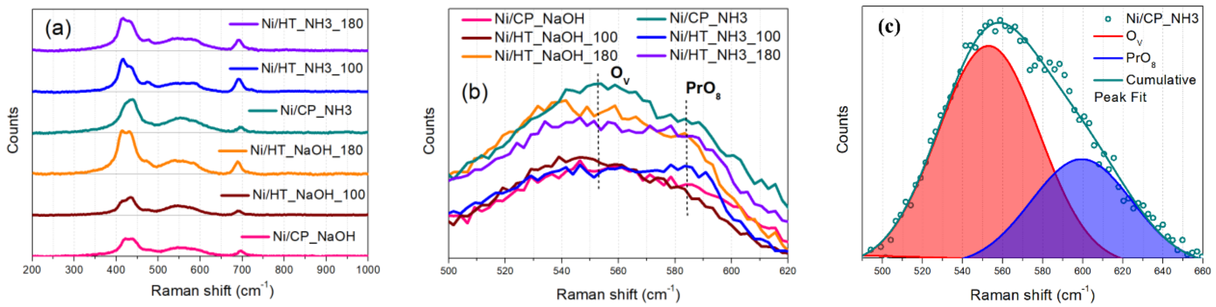

Figure S3. (**a**) Raman spectra of the reduced catalysts, (**b**) zoom at the "defects" region, and (**c**) deconvoluted Raman spectrum for the example of the Ni/CP_NH3 reduced catalyst at the "defects" region.

Table S3. I_{OV}/I_{F2G} ratios for the reduced catalysts obtained from Raman analysis.

**Catalyst**	$\mathbf{I_{OV}/I_{F2G}}$
Ni/CP_NaOH	0.50
Ni/HT_NaOH_100	0.58
Ni/HT_NaOH_180	0.32
Ni_CP_NH3	0.50
Ni/HT_NH3_100	0.32
Ni/HT_NH3_180	0.44

Table S4. XPS elemental surface concentrations, given in atomic %, and in parentheses in weight %, for the reduced catalysts.

**Catalyst**	**O (%)**	**Ni (%)**	**Ce (%)**	**Pr (%)**	**C (%)**	**Na (%)**
Ni/CP_NaOH	60.2 (24.6)	6.6 (9.9)	13.0 (46.5)	2.8 (10.1)	4.4 (1.3)	13.0 (7.6)
Ni/HT_NaOH_100	54.4 (23.5)	6.0 (9.5)	12.5 (47.2)	2.4 (9.1)	15.6 (5.1)	9.1 (5.6)
Ni/HT_NaOH_180	58.0 (25.9)	8.9 (14.6)	11.0 (43.0)	2.4 (9.4)	18.0 (6.0)	1.7 (1.1)
Ni_CP_NH3	65.2 (24.8)	9.8 (13.6)	14.8 (49.2)	3.1 (10.4)	7.1 (2.0)	n.a.
Ni/HT_NH3_100	56.8 (21.9)	12.7 (18.0)	13.9 (47.0)	2.7 (9.2)	13.8 (4.0)	n.a.
Ni/HT_NH3_180	57.4 (25.6)	14.6 (23.9)	9.6 (37.5)	1.9 (7.5)	16.5 (5.5)	n.a.

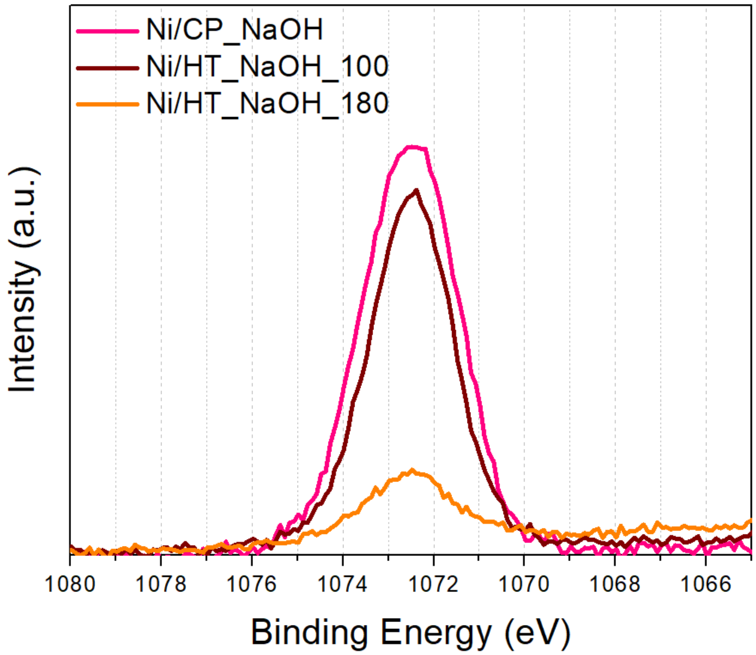

Figure S4. Na1s XPS core level spectra of the reduced catalysts whose supports were prepared using NaOH as the precipitating agent.

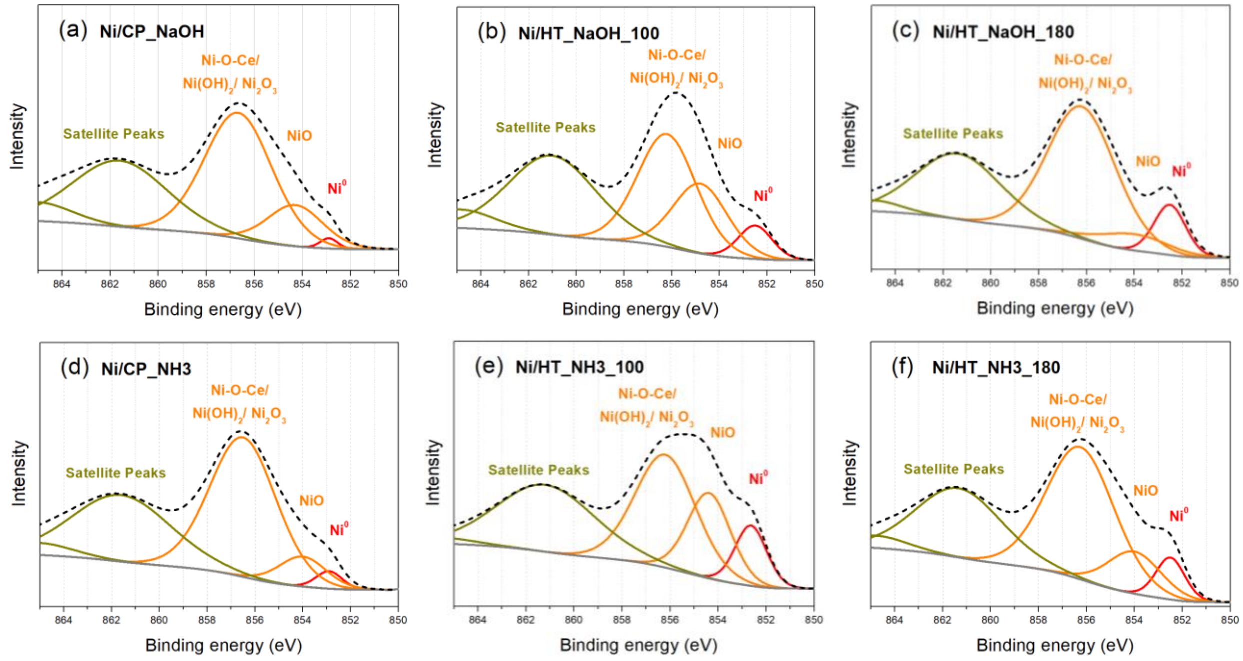

Figure S5. Peak deconvolution of the Ni2p XPS core level spectra for the reduced catalysts.

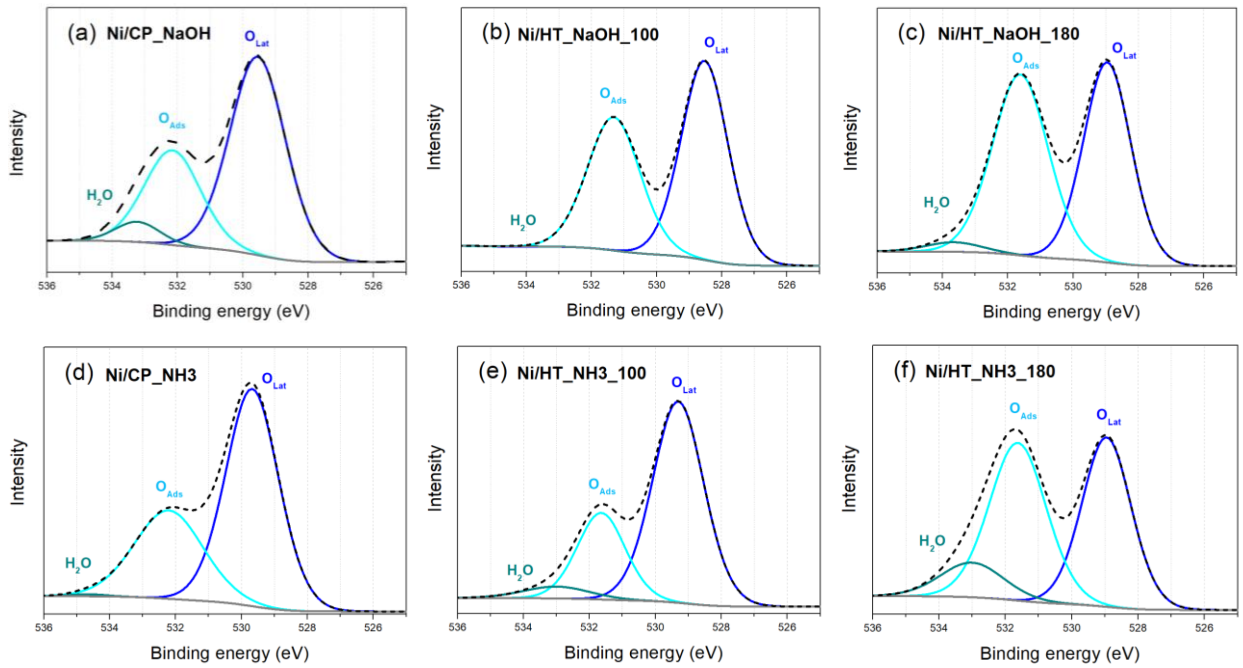

Figure S6. Peak deconvolution of the O1s XPS core level spectra for the reduced catalysts.

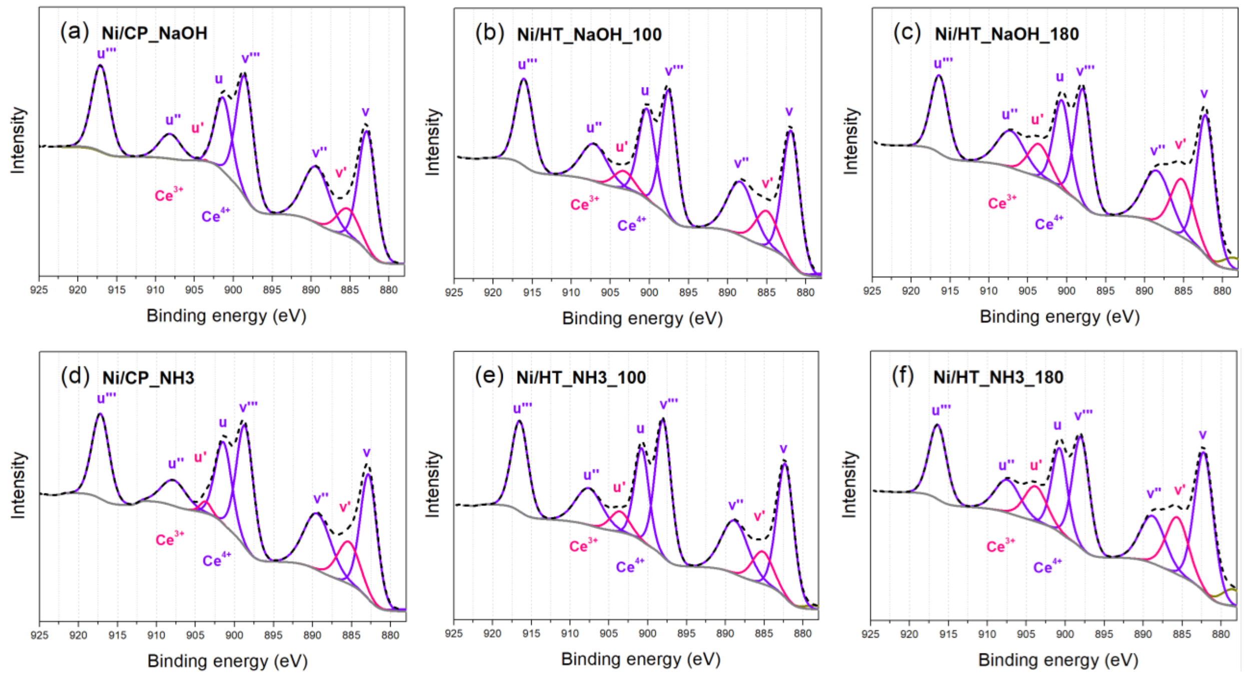

Figure S7. Peak deconvolution of the Ce3d XPS core level spectra for the reduced catalysts.

Table S5. Ratios for metallic Ni, $Ni^0/(Ni^0 + Ni^{x+})$, adsorbed oxygen species, $O_{Ads}/(O_{Ads} + O_{Lat})$, and Ce^{3+} species, $Ce^{3+}/(Ce^{3+} + Ce^{4+})$, derived from the XPS spectra following peak deconvolution.

Catalyst	$Ni^0/(Ni^0 + Ni^{x+})$ (%)	$O_{Ads}/(O_{Ads} + O_{Lat})$ (%)	$Ce^{3+}/(Ce^{3+} + Ce^{4+})$ (%)
Ni/CP_NaOH	2	38	8
Ni/HT_NaOH_100	11	43	11
Ni/HT_NaOH_180	14	51	18
Ni_CP_NH3	5	35	12
Ni/HT_NH3_100	15	29	10
Ni/HT_NH3_180	10	52	18

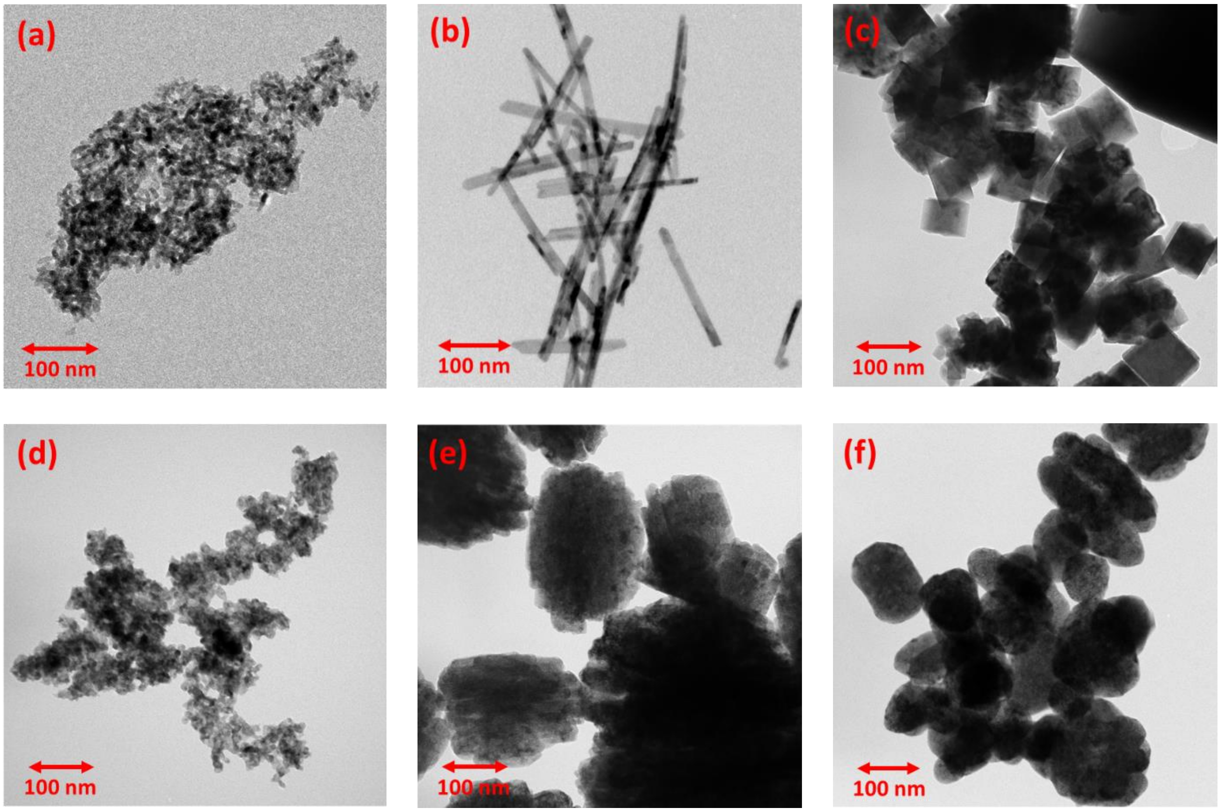

Figure S8. TEM images of the (**a**) CP_NaOH, (**b**) HT_NaOH_100, (**c**) HT_NaOH_180, (**d**) CP_NH3, (**e**) HT_NH3_100, and (**f**) HT_NH3_180 calcined metal oxide supports.

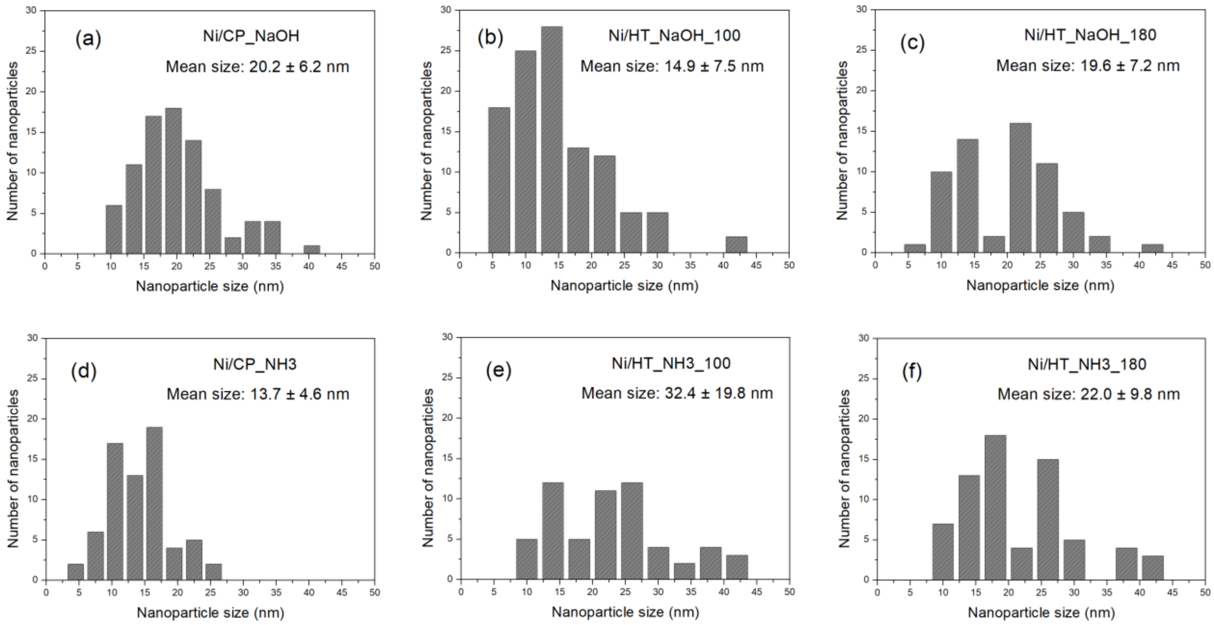

Figure S9. Ni nanoparticle size distribution histograms of the (**a**) Ni/CP_NaOH, (**b**) Ni/HT_NaOH_100, (**c**) Ni/HT_NaOH_180, (**d**) Ni/CP_NH3, (**e**) Ni/HT_NH3_100, and (**f**) Ni/HT_NH3_180 reduced catalysts, with the corresponding mean Ni nanoparticle size.

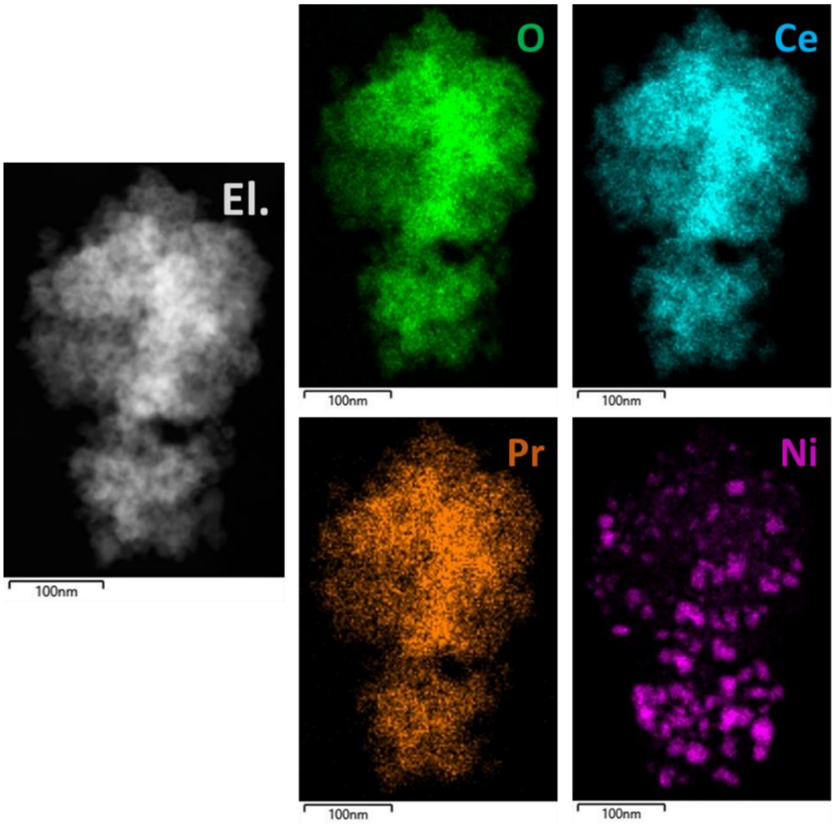

Figure S10. HAADF-STEM along with EDS elemental mapping for O, Ce, Pr, and Ni for the Ni/CP_NH3 reduced catalyst.

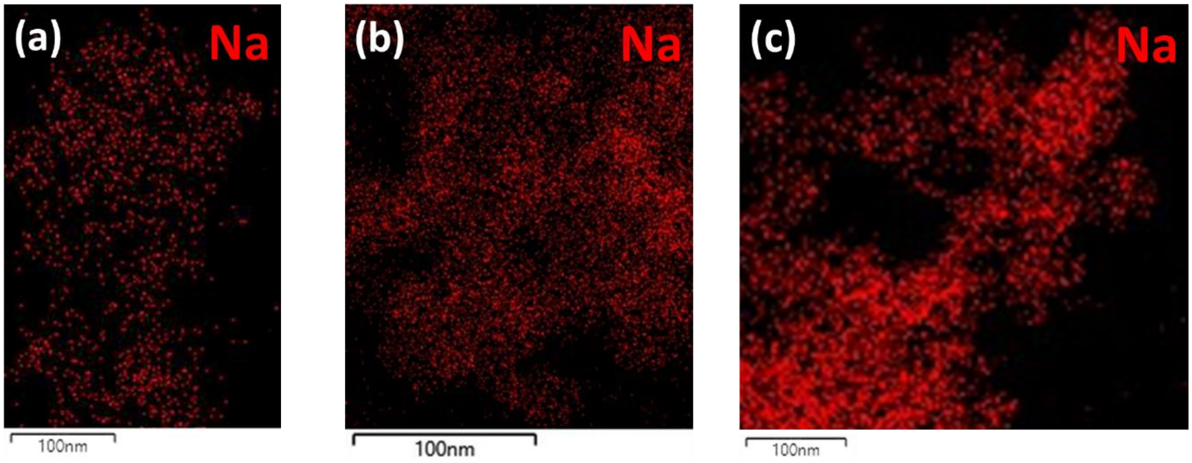

Figure S11. Na EDS elemental mapping images for the catalysts prepared with NaOH: (**a**) Ni/CP_NaOH, (**b**) Ni/HT_NaOH_100, and (**c**) Ni/HT_NaOH_180. The sites in these images correspond to those in Figure 5.

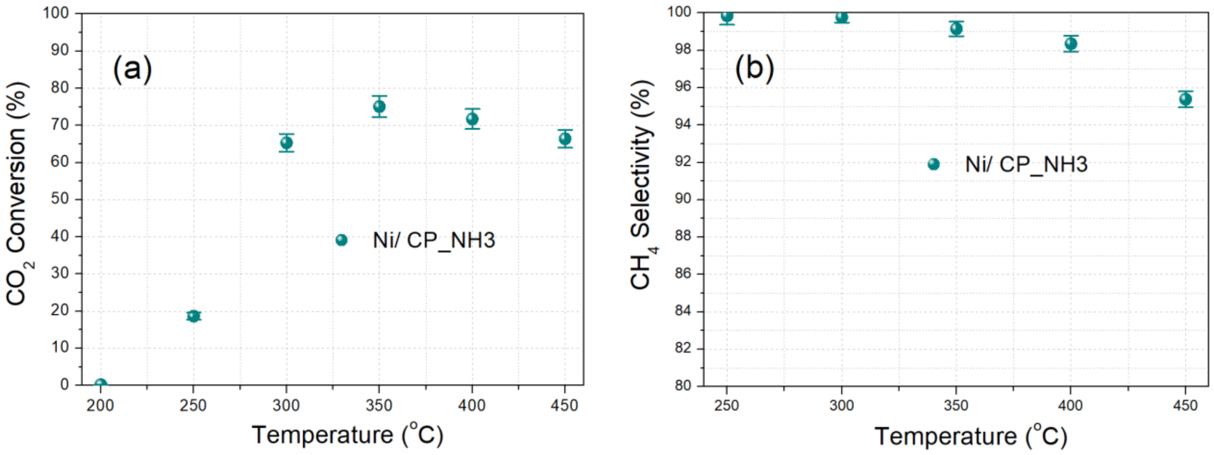

Figure S12. Average values for (**a**) CO_2 conversion and (**b**) CH_4 selectivity for the Ni/CP_NH3 catalyst from 3 different experiments, along with the corresponding error bars (Experimental Protocol #1).

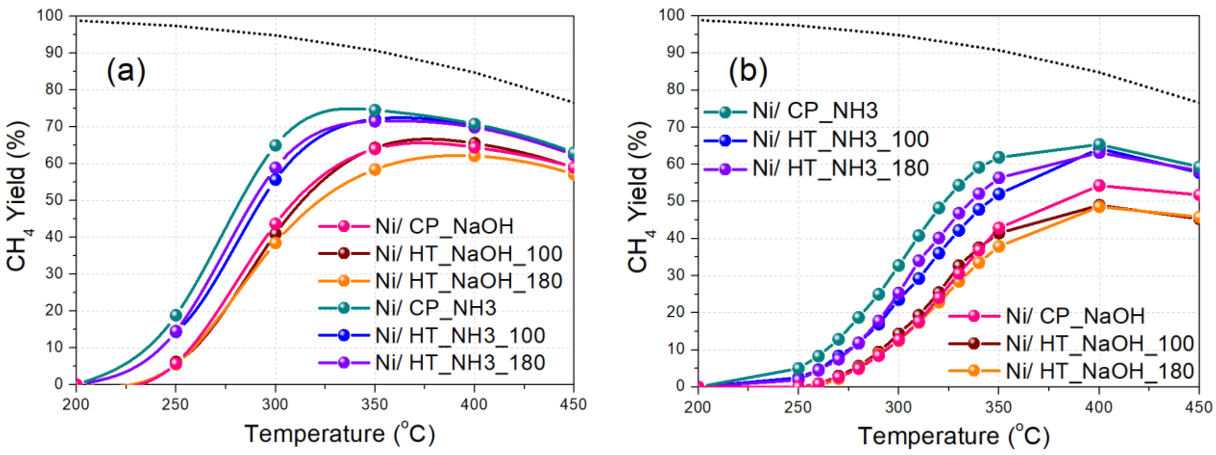

Figure S13. CH_4 yield as a function of reaction temperature; (**a**) Reaction conditions: Experimental Protocol #1; (**b**) Reaction conditions: Experimental Protocol #2. The thermodynamic equilibrium (dotted lines) is calculated via Aspen Plus (p = 1 atm and H_2:CO_2 = 4:1).

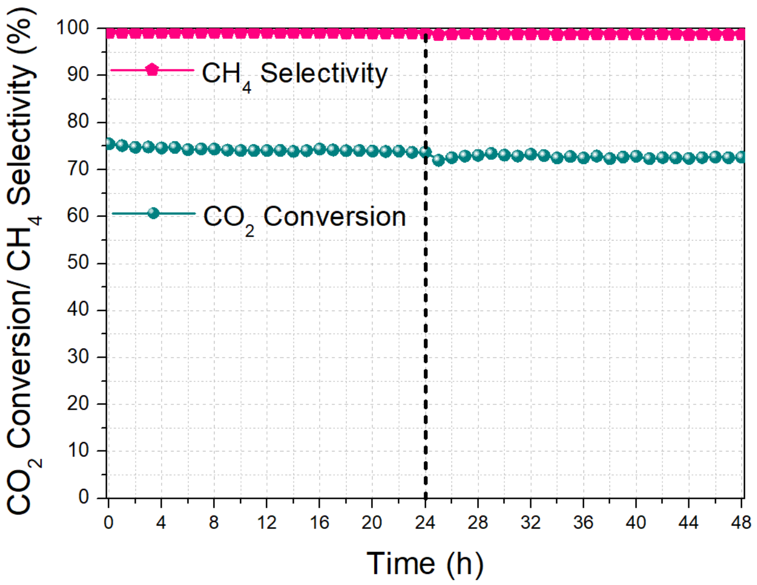

Figure S14. Time-on-stream catalytic stability for Ni/CP_NH3 at 350 ºC for 24 h, and then for an additional 24 h (48 h total) after reloading the spent catalyst in the reactor (Experimental Protocol #3). The two 24 h segments are separated via a dashed line.
